# Bromate reduction by *Shewanella oneidensis* MR-1 is mediated by dimethylsulfoxide reductase

**DOI:** 10.3389/fmicb.2022.955249

**Published:** 2022-08-30

**Authors:** Yicheng Wang, Jiale Fan, Yonglin Shen, Fan Ye, Zhiying Feng, Qianning Yang, Dan Wang, Xunchao Cai, Yanping Mao

**Affiliations:** ^1^College of Chemistry and Environmental Engineering, Shenzhen University, Shenzhen, China; ^2^Department of Gastroenterology and Hepatology, Shenzhen University General Hospital, Shenzhen, China

**Keywords:** *Shewanella oneidensis*, bromate reduction, bromate, bromide, molecular mechanism, DMSO reductase, genomic survey

## Abstract

Microbial bromate reduction plays an important role in remediating bromate-contaminated waters as well as biogeochemical cycling of bromine. However, little is known about the molecular mechanism of microbial bromate reduction so far. Since the model strain *Shewanella oneidensis* MR-1 is capable of reducing a variety of oxyanions such as iodate, which has a high similarity to bromate, we hypothesize that *S. oneidensis* MR-1 can reduce bromate. Here, we conducted an experiment to investigate whether *S. oneidensis* MR-1 can reduce bromate, and report bromate reduction mediated by a dimethylsulfoxide reductase encoded with *dmsA*. *S. oneidensis* MR-1 is not a bromate-respiring bacterium but can reduce bromate to bromide under microaerobic conditions. When exposed to 0.15, 0.2, 0.25, 0.5, and 1 mM bromate, *S. oneidensis* MR-1 reduced bromate by around 100, 75, 64, 48, and 23%, respectively, within 12 h. *In vivo* evidence from gene deletion mutants and complemented strains of *S. oneidensis* MR-1 indicates that MtrB, MtrC, CymA, GspD, and DmsA are involved in bromate reduction, but not NapA, FccA, or SYE4. Based on our results as well as previous findings, a proposed molecular mechanism for bromate reduction is presented in this study. Moreover, a genomic survey indicates that 9 of the other 56 reported *Shewanella* species encode proteins highly homologous to CymA, GspD, and DmsA of *S. oneidensis* MR-1 by sequence alignment. The results of this study contribute to understanding a pathway for microbial bromate reduction.

## Introduction

Bromate (${\text{BrO}}_{3}^{-}$), an oxyanion of bromine, has been classified by the World Health Organization as a possible human carcinogen (WHO, 2011). Bromate contamination has been detected in various environments (e.g., drinking water, wastewater, surface water, and groundwater) posing human health risks (Butler et al., 2005; Jahan et al., 2021). Microbial bromate reduction is a promising method for bioremediating bromate-contaminated waters, and has attracted extensive attention (Zhong et al., 2018; Lv et al., 2019; Liu et al., 2020; Jahan et al., 2021).

To date, there are only a few reports on bromate reduction by isolates (e.g., *Rhodococcus* sp. Br-6, *Dechloromonas* sp. PC1, *Klebsiella variicola* Glu3, and *Shewanella decolorationis* Ni1-3) (Davidson et al., 2011; Tamai et al., 2016; Jahan et al., 2021; Wang D. et al., 2022; Wang Y. et al., 2022). *Rhodococcus* sp. Br-6 reduced bromate to bromide under transition conditions (from aerobic to anaerobic conditions), and that reaction was significantly dependent on both ferric iron and a redox mediator, 2,6-dichloroindophenol (Tamai et al., 2016). In addition, terminal reductases purified from bacteria, such as (per)chlorate reductase (PcrA) (Kengen et al., 1999), chlorate reductase (ClrA) (Thorell et al., 2003), nitrate reductase (NarG) (Morpeth and Boxer, 1985; Maria Martinez-Espinosa et al., 2015), selenate reductase (SerA) (Ridley et al., 2006), and trimethylamine-N-oxide reductase (TorA) (Shimokawa and Ishimoto, 1979), have only shown bromate-reducing activity *in vitro*, but whether they can mediate bromate reduction *in vivo* remains unclear. So far, little is known about microbial bromate reduction because of limited number of available isolates and paucity of information for key genes involved in that reaction (Jahan et al., 2021). Moreover, a variety of isolates can reduce oxidative oxyanions [i.e., nitrate and Cr(VI)] in the presence of oxygen (Pradhan et al., 2017; Huang et al., 2020; Yang et al., 2020; Karimi-Maleh et al., 2021; Zhang et al., 2021), but the (micro)aerobic reduction of bromate by pure cultures is poorly understood at present.

*Shewanella* species are facultative anaerobic bacteria well-known for their remarkable respiratory diversity (Hau and Gralnick, 2007; Fredrickson et al., 2008; Lemaire et al., 2020). The *Shewanella* genus currently includes around 70 species that are widely distributed in aquatic environments such as freshwater and marine sediments around the world (Lemaire et al., 2020). Knowledge of the respiratory diversity of *Shewanella* species is mainly derived from the model strain *Shewanella oneidensis* MR-1, which can reduce diverse oxyanions including iodate, sulfite, nitrate, U(VI), Cr(VI), and selenite (Shirodkar et al., 2011; Li et al., 2014; Beblawy et al., 2018; Lemaire et al., 2020; Vettese et al., 2020; Guo et al., 2022; Shin et al., 2022). Bromate is a halogen oxyanion with a molecular structure and chemical properties similar to iodate. Additionally, a recent study shows that *S. decolorationis* Ni1-3 can perform bromate reduction, and that its genome shares an average nucleotide identity (ANI) of 85% with *S. oneidensis* MR-1 (Wang Y. et al., 2022). Taken together, we anticipate that the model strain *S. oneidensis* MR-1 is able to reduce bromate. Based on this hypothesis, we intend to address what enzymes mediate bromate reduction by *S. oneidensis* MR-1.

Previous studies suggest that nitrate reductase might be responsible for bromate reduction, and FccA (periplasmic fumarate reductase) was shown to mediate selenite reduction by *S. oneidensis* MR-1 (Hijnen et al., 1995, 1999; Li et al., 2014). Hence, we hypothesize that NapA (periplasmic nitrate reductase) or FccA may contribute to bromate reduction by *S. oneidensis* MR-1. As a powerful oxidant, bromate can induce oxidative stress in cells (Ahmad et al., 2015). SYE4 sequences of *S. oneidensis* MR-1 and the NemA [Cr(VI) reductase of *Escherichia coli*] share an identity of 42%, both belonging to the old yellow enzyme (a NAPDH oxidoreductase) family, and SYE4 has been reported to be induced under oxidative stress (Brigé et al., 2006; Thatoi et al., 2014). Moreover, recent evidence suggests that *sye4* of *S. decolorationis* Ni1-3 was highly induced in response to bromate (Wang Y. et al., 2022). Therefore, we also hypothesize that SYE4 may contribute to bromate reduction by *S. oneidensis* MR-1.

This study aims (1) to test the hypothesis that *S. oneidensis* MR-1 can reduce bromate, (2) to explore the bromate reductase of *S. oneidensis* MR-1, and (3) to identify whether all *Shewanella* species possess key proteins related to bromate reduction. The experimental strategy consists of the following steps: (1) batch cultivation under both anaerobic and microaerobic conditions to test the bromate-reducing capacity of *S. oneidensis* MR-1, (2) construction of in-frame deletion mutants and complemented strains and subsequent measurement of bromate-reducing capacity, and (3) identification of a homologous protein required for bromate reduction among the other 56 *Shewanella* species.

## Materials and methods

### Bacterial strains, plasmids, and culture conditions

The bacterial strains and plasmids used in this study are listed in Table 1. *S. oneidensis* and *E. coli* strains were routinely cultured aerobically at 30 and 37°C in lysogeny broth (LB) (10 g/L NaCl, 5 g/L yeast extract, and 10 g/L tryptone). When required, the LB medium was supplemented with chemicals at the following concentrations: 2,6-diaminopimelicacid 50 μg/ml, gentamycin 15 μg/ml, and kanamycin 50 μg/ml. Bromate reduction by *S. oneidensis* strains was performed under both anaerobic and microaerobic conditions in a bromate reduction (BR) medium (pH = 7.2), which contained 2.24 g sodium lactate (20 mM), 1.2 g Na_2_HPO_4_, 0.8 g KH_2_PO_4_, 1 g (NH_4_)_2_HPO_4_, 0.1 g yeast extract, 0.1 g tryptone, 10 ml vitamin solution (Wolin et al., 1963), and 1 ml trace element solution per liter (Supplementary Table 1).

**TABLE 1:** Bacterial strains and plasmids used in this study.

Strain or plasmid	Description	Source or references
**Strains**		
*E. coli* WM3064	Donor strain for conjugation; Δ*dapA*	Shi et al., 2013
***S. oneidensis* strains**		
MR-1	Wild type	CCTCC AB 2013238
Δ*napA*	In-frame *napA* deletion mutant	This study
Δ*fccA*	In-frame *fccA* deletion mutant	This study
Δ*sye4*	In-frame *sye4* deletion mutant	This study
Δ*cymA*	In-frame *cymA* deletion mutant	This study
Δ*gspD*	In-frame *gspD* deletion mutant	This study
Δ*mtrB*	In-frame *mtrB* deletion mutant	This study
Δ*mtrC*	In-frame *mtrC* deletion mutant	This study
Δ*dmsA*	In-frame *dmsA* deletion mutant	This study
**Plasmids**		
pHGM01	Gm*^R^*; Cm*^R^*; Ap*^R^*; *sacB*; Ori-R6K; suicide plasmid for generating in-frame deletions	Jin et al., 2013
pHGE	pHGE-P*_*tac*_*, Km*^R^*, IPTG-inducible expression plasmid	Shi et al., 2013
pHGE-*cymA*	Km*^R^*; plasmid for expressing the wild-type *cymA*	This study
pHGE-*dmsA*	Km*^R^*; plasmid for expressing the wild-type *dmsA*	This study

IPTG, isopropyl-β-D-1-thiogalactopyranoside.

### Bromate reduction

The bromate reduction by *S. oneidensis* strains was conducted at 30°C. *S. oneidensis* MR-1 was incubated aerobically in the LB medium for ∼12 h, and cell pellets were collected and washed with phosphate buffered solution (PBS) and transferred into a serum bottle that contained 100 ml BR medium, and the bromate was added to the culture before aeration. Oxygen was purged with high-purity nitrogen gas for 10 min, and the culture was incubated anaerobically and shaken at 150 rpm. For microaerobic bromate reduction, *S. oneidensis* strains were incubated aerobically in the LB medium for ∼12 h, and cell pellets were collected and washed once with PBS and transferred into a 250-ml Erlenmeyer flask containing 100 ml BR medium at an initial OD_600_ (optical density at 600 nm) of 0.12. The culture was added with bromate and incubated and shaken at 150 rpm.

### Mutagenesis and complementation

In-frame markerless deletion strains were constructed by seamless cloning and SacB-based counterselection as described by Jin et al. (2013). Briefly, two fragments (500–1,000 bp in length) flanking the target gene and linearized pHGM01 were recombined using a Hi-Fusion Cloning Mix V2 kit (Monad, China) according to the manufacturer’s instructions. The resulting plasmids were maintained in *Escherichia coli* WM3064 and subsequently transferred into *S. oneidensis* strains by conjugation. Verified transconjugants were grown in LB medium without NaCl and subsequently plated on LB agar plates supplemented with 10% sucrose. Sucrose-resistant and gentamicin-sensitive colonies were screened by PCR for the intended deletion. Deletion mutants were then verified by Sanger sequencing. For complementation of genes, a fragment containing the gene of *S. oneidensis* MR-1 wild type was generated by PCR and cloned into pHGE. After verification by Sanger sequencing, the resultant plasmids were transferred into relevant strains by conjugation. The primers used for mutagenesis and complementation are listed in Supplementary Table 2.

### Determination of biomass and chemical assays

The OD_600_ of the cultures was detected using a Synergy HTX multi-mode plate reader (BioTek, United States). For total protein quantification, cell pellets were collected by centrifugation and resuspended in 0.85% (w/v) NaCl solution, and cells were disrupted by sonication at 200 W for 5 min. Protein concentrations were then determined using the method described by Bradford (1976). Bromate and bromide concentrations were determined by the 883 Basic IC plus ion chromatograph (Metrohm, Switzerland) using a Metrosep A Supp 7-250/4.0 column and an eluent consisting of 3.6 mmol/L Na_2_CO_3_ with 2% (v/v) acetonitrile at 0.7 ml/min. The dissolved oxygen (DO) concentration was measured using a portable JPB-607A DO meter (REX, China). The pH value of the cultures was determined using a LAQUAtwin pH-11 meter (Horiba, Japan).

### Sequence alignment and phylogenetic analysis

Reference genomes of 57 species of *Shewanella* genus (*S. oneidensis* MR-1 included) were downloaded from NCBI database (Supplementary Table 3). In this study, a database was constructed using the CymA, GspD, and DmsA protein sequences of *S. oneidensis* MR-1. Based on BLAST (Camacho et al., 2009) alignment, we identified whether the other 56 *Shewanella* species contain homologous protein sequences of CymA, GspD, and DmsA. Positive alignments were improved based on the criteria (identity > 70%, query coverage > 80%, and *e*-value < 10^–5^) described by Assis et al. (2017). The MEGA X software (Kumar et al., 2018) was used to align the identified homolog sequences to DmsA with those of other DMSO reductases and construct a phylogenetic tree based on the neighbor-joining method (Naruya and Masatoshi, 1987). The phylogenetic tree was visualized using the iTOL software (Letunic and Bork, 2021).

### Other analysis

A Mann–Whitney U test was conducted using the GraphPad Prism software for pairwise comparisons of groups.

## Results

### Bromate reduction by *Shewanella oneidensis* MR-1

To test whether *S. oneidensis* MR-1 could be a bromate-respiring bacterium, a set of batch cultivation was performed with bromate as the sole electron acceptor under anaerobic conditions. It was found that only about 7% (24 h) of bromate was reduced with the dosage of 1 mM bromate, and that almost no bromate reduction occurred within 24 h when the dosage of 2 mM bromate was used (Supplementary Figure 1A). Moreover, the biomass of *S. oneidensis* MR-1 did not increase in the presence of bromate under anaerobic conditions (Supplementary Figure 1B).

**FIGURE 1:**
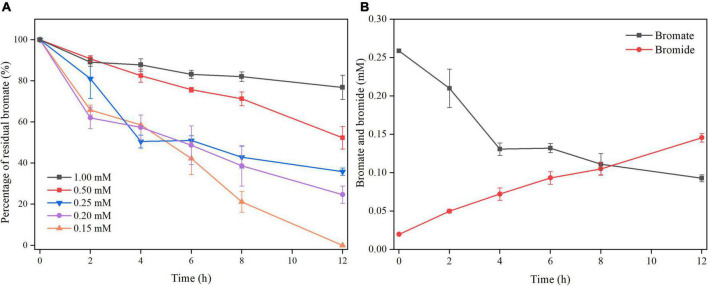
Microaerobic bromate reduction by *S. oneidensis* MR-1. **(A)** The strain was cultured with the dosage of bromate at 0.15, 0.2, 0.25, 0.5, and 1 mM. **(B)** The strain was cultured with the dosage of bromated at 0.25 mM. Error bars represent standard deviations of triplicate samples.

Another set of batch cultivation was carried out to test whether *S. oneidensis* MR-1 could reduce bromate under microaerobic conditions. Obvious bromate reduction was observed within 12 h under microaerobic conditions, and with increase in bromate concentration, the bromate-reducing efficiency of *S. oneidensis* MR-1 was decreased (Figure 1A). Bromate was completely reduced by *S. oneidensis* MR-1 with the dosage of 0.15 mM, and when the dosage of bromate was 0.2, 0.25, 0.5, and 1 mM, the bromate-reducing efficiencies (12 h) reached around 75, 64, 48, and 23%, respectively. Under the microaerobic conditions of this study, the DO concentration (0–12 h) in the culture was maintained at around 3.5 mg/L (Supplementary Figure 2). Besides, measurable growth of *S. oneidensis* MR-1 was observed under microaerobic conditions, and bromate at these concentrations (0.15–1 mM) appeared to neither promote nor inhibit the growth of *S. oneidensis* MR-1 (Supplementary Figure 3). The bromide concentration increased as the bromate concentration was decreased, indicating that bromate was eventually reduced to bromide (Figure 1B).

To demonstrate whether microaerobic bromate reduction is dependent on biological process, two control experiments were performed with the dosage of of 0.25 mM bromate. Only about 6% of bromate was reduced using heat-killed cells (Supplementary Figure 4), but no bromide was detected. In the absence of lactate, almost no bromate reduction occurred (Supplementary Figure 4). The above results suggest that bromate reduction is dependent on the metabolism of *S. oneidensis* MR-1.

### Bromate-reducing capacities of Δ*napA*, Δ*fccA*, and Δ*sye4* mutants

**FIGURE 2:**
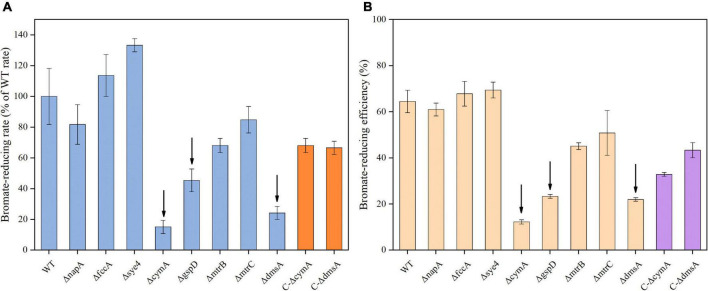
Microaerobic reduction of bromate at 0.25 mM by the *S. oneidensis* MR-1 wild type (WT), in-frame deletion mutants (Δ*napA*, Δ*fccA*, Δ*sye4*, Δ*cymA*, Δ*gspD*, Δ*mtrB*, Δ*mtrC*, and Δ*dmsA*), and complemented strains (C-Δ*cymA* and C-Δ*dmsA*, bromate reduction was performed in the presence of 0.2 mM IPTG). **(A)** Bromate-reducing rate (μMh^– 1^ mg protein^– 1^) was calculated from the first 2 h of incubation and normalized to total protein concentration. **(B)** Bromate-reducing efficiency represents the proportion of reduced bromate (at 12 h) in the initial bromate. Error bars represent standard deviations of triplicate samples.

To identify the key reductase involved in microaerobic bromate reduction, a number of in-frame deletion mutants derived from the *S. oneidensis* MR-1 wild type (WT) were constructed, and their bromate-reducing capacities were evaluated at an identical bacterial concentration (OD_600_ = 0.12). The putative bromate reductase-encoding genes (*napA, fccA*, and *sye4*) described in the introduction were first knocked out. However, compared to WT, Δ*napA*, Δ*fccA*, and Δ*sye4* all showed no significant (*p* ≥ 0.2) difference in bromate-reducing rate and efficiency (Figures 2A,B). The results suggest that NapA, FccA, and SYE4 are not required in microaerobic bromate reduction by *S. oneidensis* MR-1.

### Involvement of the terminal reductase located in the outer membrane

To determine whether cytoplasmic, periplasmic, or extracellular reductase mediates microaerobic bromate reduction, Δ*cymA* and Δ*gspD* were constructed. Interestingly, the deletion of *cymA* or *gspD* severely impaired the bromate-reducing capacity of *S. oneidensis* MR-1. Compared to the WT, the bromate-reducing rate and efficiency of Δ*cymA* were decreased by 85 and 52%, respectively; correspondingly those of Δ*gspD* were decreased by 55 and 41%, respectively (Figures 2A,B). The results indicate that CymA, GspD, and outer membrane proteins are responsible for bromate reduction. Furthermore, we constructed three in-frame deletion mutants (i.e., Δ*mtrB*, Δ*mtrC*, and Δ*dmsA*) to identify which outer membrane protein is required for bromate reduction. By deletion of *mtrB* or *mtrC*, *S. oneidensis* MR-1 exhibited a slight defect in bromate-reducing capacity (Figures 2A,B). With *dmsA* deleted, the bromate-reducing ability of *S. oneidensis* MR-1 was impaired to a degree close to that of Δ*cymA* and greater than that of Δ*gspD*. Moreover, the bromate-reducing capacities of complemented strains C-Δ*cymA* and C-Δ*dmsA* were recovered relative to the gene deletion mutant strains (i.e., Δ*cymA* and Δ*dmsA*).

### Identification of homologous proteins and phylogenetic relationship

**FIGURE 3:**
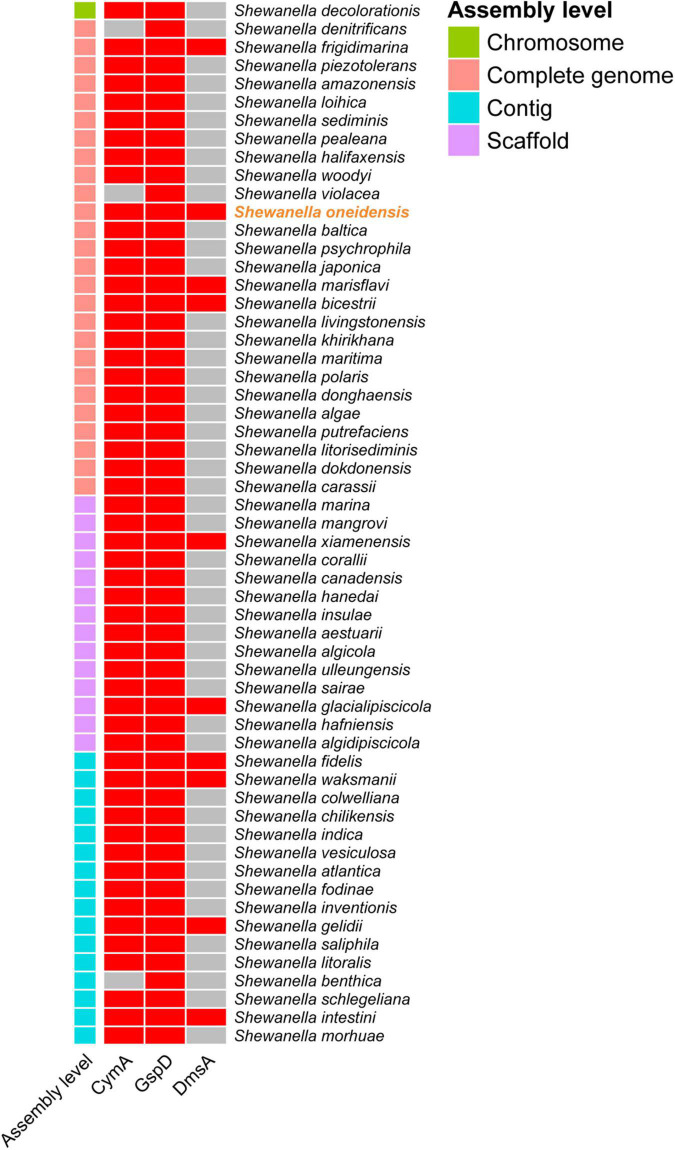
Homologous protein identification of *Shewanella* species. Cells colored in red or gray indicate if the specific protein is identified or not, respectively.

**FIGURE 4:**
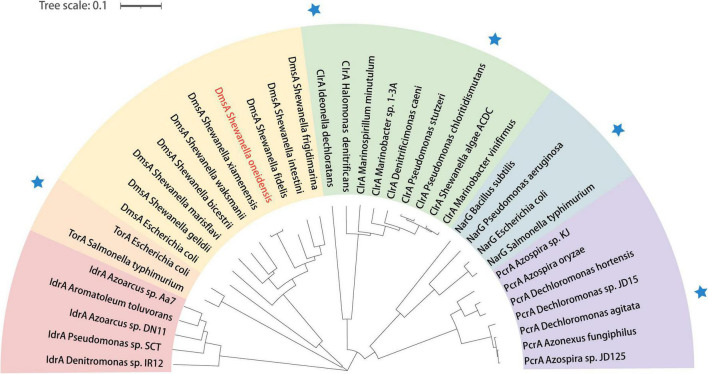
Phylogenetic analysis of DmsA of *Shewanella* species in relation to other reductases in the DMSO reductase family. The phylogenetic tree is constructed using the neighbor-joining method, and the bootstrap values of all nodes in the tree are not less than 50%. Blue star indicates that the protein has exhibited bromate-reducing activity *in vitro*. IdrA, iodate reductase; TorA, trimethylamine-oxide reductase; DmsA, DMSO reductase; ClrA, chlorate reductase; PcrA, perchlorate reductase.

To explore whether all the *Shewanella* species possess key proteins related to bromate reduction, protein sequence alignment was performed. According to the alignment standard of the present study, it was found that 9 of the 56 other *Shewanella* species with a whole-genome sequence possess homologs to DmsA, GspD, and CymA of *S. oneidensis* MR-1 (Figure 3). In order to explore the phylogenetic relationship between the DmsA sequences of *Shewanella* species (the DmsA sequence of *Shewanella glacialipiscicola* was eliminated because of incompleteness) and 28 other DMSO reductase sequences, a phylogenetic tree was constructed. As shown in Figure 4, the DmsA of *Shewanella* species and *E. coli* belongs to the same major clade, which is different from the other DMSO reductases, including PcrA, ClrA, NarG, and TorA (these reductases have shown bromate-reducing activity *in vitro*), and the phylogenetic relationship of this clade and the TorA clade is the closest.

## Discussion

*Shewanella oneidensis* MR-1 did not exhibit the characteristic of bromate-respiring under anaerobic conditions (Supplementary Figures 1A,B), indicating that *S. oneidensis* MR-1 may not respire a high concentration of bromate under the anaerobic conditions of this study. However, according to previous findings (Toporek et al., 2019; Guo et al., 2022; Shin et al., 2022), when *S. oneidensis* MR-1 has sufficient biomass, it is still possible to perform anaerobic reduction of bromate at low concentrations. As expected, it was found that *S. oneidensis* MR-1 and the bromate-reducing bacterium *S. decolorationis* Ni1-3 have a similar bromate-reducing ability, and that both of them can reduce bromate to bromide with high efficiency (Wang Y. et al., 2022; Figures 1A,B). *S. oneidensis* MR-1 can grow at relatively high bromate concentrations under microaerobic conditions, which is similar to a previous study on perchlorate-reducing bacteria that some halophilic bacteria can grow in the presence of perchlorate at as high as 0.4 M under aerobic conditions (Oren et al., 2014). Although there are several reports on reduction of bromate under (micro)aerobic conditions in the biologically active carbon (BAC) filter, no available isolate capable of (micro)aerobic bromate reduction has been isolated (Kirisits and Snoeyink, 1999; Kirisits et al., 2001, 2002; Liu et al., 2012). Recently, a transcriptome analysis has provided insights into the tolerance and aerobic reduction of *S. decolorationis* Ni1-3 to bromate, but no bromate reductase has been identified (Wang Y. et al., 2022).

Previous studies have shown that dissimilatory nitrate reductase (NarG) can reduce bromate *in vitro* (Morpeth and Boxer, 1985; Maria Martinez-Espinosa et al., 2015). *S. oneidensis* MR-1 has only one dissimilatory nitrate reductase, NapA, which is homologous to NarG. However, the results suggest that NapA is not required for microaerobic bromate reduction by *S. oneidensis* MR-1. This finding is similar to a previous study that NapA is not involved in iodate reduction by *S. oneidensis* MR-1 (Mok et al., 2018). The *in vivo* evidence from the mutants (Δ*fccA* and Δ*sye4)* also disproves our hypothesis that FccA and SYE4 are involved in microaerobic bromate reduction by *S. oneidensis* MR-1. Membrane-anchored CymA is a key component of the electron transport chain in the extracellular and periplasmic spaces (McMillan et al., 2012). GspD is an important protein in the type II secretion system, which transports extracellular terminal reductases (e.g., MtrC, OmcA, and DmsA) to the outer membrane surface (Rondelet and Condemine, 2013). In the present study, the bromate-reducing ability of Δ*cymA* and Δ*gspD* was severely impaired, suggesting that CymA, GspD, and outer membrane proteins are involved in bromate reduction. It should be noted that the bromate-reducing ability of Δ*cymA*, Δ*gspD*, and Δ*dmsA* is not completely lost, and whether TorA located in the periplasm is responsible for small partial bromate reduction needs to be further determined.

MtrC and OmcA, complexed together in a ratio of 1:2, are typical extracellular terminal reductases of *S. oneidensis* MR-1, which can reduce U(VI), Cr(VI), V(V), and Tc(VII) (Beblawy et al., 2018). MtrAB is responsible for transferring electron to MtrC (Beblawy et al., 2018). The results of this study rule out the possibility that MtrC is the major terminal bromate reductase, but that MtrCAB can contribute to microaerobic reduction of bromate. The purified reductases of bacteria (i.e., PcrA, ClrA, NarG, SerA, and TorA) with bromate-reducing activity all belong to the DMSO reductase family (Miralles-Robledillo et al., 2019). DmsEFAB, the complex protein of *S. oneidensis* MR-1, has been proved to mediate the dissimilatory reduction of DMSO and the extracellular reduction of iodate (Gralnick et al., 2006; Guo et al., 2022; Shin et al., 2022). DmsA is located in the outer membrane and is the catalytic subunit; it also belongs to the DMSO reductase family (Gralnick et al., 2006). The *in vivo* evidence from the present study indicates that the terminal reductase DmsA mediates microaerobic bromate reduction by *S. oneidensis* MR-1, and that both CymA and GspD are also required in that process. Previous studies have shown that the DMSO reductase of MR-1 belongs to the anaerobic respiration system, but it can be expressed under aerobic conditions, although its expression level is less than that under anaerobic conditions (Gralnick et al., 2006). In this study, the cultures were not sparging with air or oxygen, shaking was not violent, and microaerobic or anoxic zones were easily formed in the cultures. Thus, the DMSO reductase could be expressed and perform a limited function. In addition, when the cells of *S. oneidensis* MR-1 get into the stationary phase, oxygen is not the preferred electron acceptor; other electron acceptors such as nitrite can be respired (Dong et al., 2012). Similarly, *S. oneidensis* MR-1 quickly got into the stationary phase (Supplementary Figure 3), so that the DMSO reductase might be available for extracellular reduction of bromate during that time.

**FIGURE 5:**
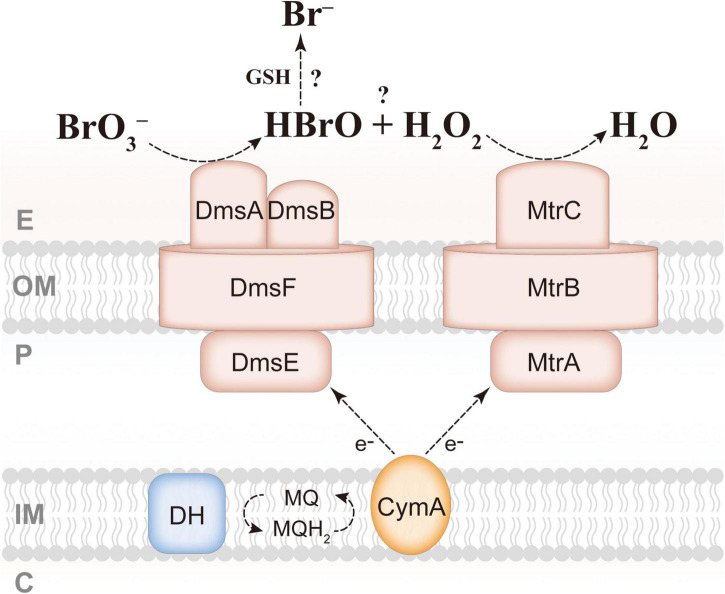
Proposed mechanism for extracellular reduction of bromate by *S. oneidensis* MR-1. GSH, reduced glutathione; E, extracellular space; OM, outer membrane; P, periplasm; IM, inner membrane; C, cytoplasm; DH, dehydrogenase; MQ, menaquinone.

It is well-known that respiratory reductases belonging to the DMSO reductase family use molybdenum as a cofactor and catalyze two-electron-transferring reactions, such as perchlorate → chlorate, chlorate → chlorite, selenate → selenite, nitrate → nitrite, and DMSO → dimethyl sulfide (DMS) (McEwan et al., 2002; Sparacino-Watkins et al., 2014; Miralles-Robledillo et al., 2019), whereas the final product of microaerobic bromate reduction by *S. oneidensis* MR-1 is bromide, and the valence of bromine is from positive hexavalent to negative monovalent, requiring six electrons. We therefore assume that there must be an intermediate, bromite, or hypobromous acid, in microaerobic bromate reduction by *S. oneidensis* MR-1. A recent study has shown that the DmsEFAB of *S. oneidensis* MR-1 is responsible for the reduction of iodate to hypoiodous acid while producing hydrogen peroxide, and that MtrCAB is involved in scavenging hydrogen peroxide, which then facilitates iodate reduction by *S. oneidensis* MR-1 (Guo et al., 2022). In addition, when *mtrCAB* was knocked out, there were still other reactive oxygen species scavengers (ROSSs) (i.e., catalases and peroxidases) that can replace MtrCAB to complete the reduction of hydrogen peroxide in *S. oneidensis* MR-1 (Guo et al., 2022). As mentioned in the introduction, bromate and iodate are quite similar, and considering that the results of this study are also consistent with those of iodate reduction by *S. oneidensis* MR-1, hypobromous acid is very likely to be the intermediate of bromate reduction. The final product of iodate reduction by *S. oneidensis* MR-1 is iodide, but how the intermediate hypoiodous acid is decomposed remains unclear (Guo et al., 2022). The intermediate of (per)chlorate (halogen oxyanions) reduction, chlorite, is decomposed into chloride and oxygen by chlorite dismutase (Cld); thus (per)chlorate-respiring bacteria can utilize high concentrations of (per)chlorate as the sole electron acceptor to gain energy for growth (Youngblut et al., 2016). Besides, the iodate-respiring bacterium *Pseudomonas* sp. SCT also has a Cld-like protein, which may reduce the intermediate of iodate reduction, hypoiodous acid, to iodide and oxygen (Yamazaki et al., 2020). *S. oneidensis* MR-1 has no protein homologous to Cld, supporting the idea that *S. oneidensis* MR-1 is not a bromate-respiring bacterium, and that the possible intermediate hypobromous acid may be scavenged by abiotic reaction. Hypobromous acid and hypochlorous acid share a high similarity, and hypochlorous acid can react with the antioxidant reduced glutathione (GSH) to form chloride (Winterbourn and Brennan, 1997; Fang and Dehaen, 2021); thus, hypobromous acid may also be reduced to bromide by GSH. GSH is ubiquitous in proteobacteria, and *S. oneidensis* MR-1 is found to possess two genes, *gsh*A and *gsh*B, necessary for synthesis of GSH (Masip et al., 2006). Based on these findings, a molecular mechanism was proposed for microaerobic bromate reduction by *S. oneidensis* MR-1 (Figure 5). It is assumed that bromate is reduced to hypobromous acid and hydrogen peroxide by DmsEFAB. Subsequently, hypobromous acid is reduced to bromide by GSH, and hydrogen peroxide is reduced to H_2_O by MtrCAB or other ROSSs; these two processes may limit the bromate-reducing ability of *S. oneidensis* MR-1. Future research should focus on determining whether hypobromous acid and hydrogen peroxide are the intermediates and the role of GSH in bromate reduction.

The result of the sequence alignment indicates that 9 *Shewanella* species (i.e., *S. frigidimarina*, *S. marisflavi*, *S. bicestrii*, *S. xiamenensis*, *S. glacialipiscicola*, *S. fidelis*, *S. waksmanii*, *S. gelidii*, and *S. intestini*) most likely possess a bromate-reducing capacity and may play important roles in biogeochemical cycling of bromine. The result of the phylogenetic analysis shows that the DmsA of *Shewanella* species and other DMSO reductases belong to different major clades. Interestingly, the DMSO reductases of the four different major clades exhibit a bromate-reducing activity. In the future, it would be interesting to explore whether the catalytically active centers of the DMSO reductases are quite similar, and how many of the remaining DMSO reductases have a bromate-reducing activity.

In summary, we demonstrated that *S. oneidensis* MR-1 can effectively reduce bromate under microaerobic conditions, and this process is mediated by the extracellular terminal reductase DmsA. The microbial reduction process of bromate also requires membrane-anchored CymA and the type II protein secretion system. Moreover, by protein sequence alignment, it was found that a total of 9 *Shewanella* species possess homologs to DmsA, GspD, and CymA of *S. oneidensis* MR-1. The results of this study provide new insights into the molecular mechanism of microbial bromate reduction and indicate that *Shewanella* strains may play roles in biogeochemical cycling of bromine.

## Data availability statement

The original contributions presented in this study are included in the article/Supplementary material, further inquiries can be directed to the corresponding author.

## Author contributions

YW, JF, YS, FY, ZF, and QY performed the experiments. YW wrote the manuscript. JF, DW, XC, and YM reviewed and revised the manuscript. YM supervised the whole study. All authors read and approved the final version of the manuscript.
