# Supplementary material for: Bromate reduction by Shewanella oneidensis MR-1 is mediated by dimethylsulfoxide reductase
Source: Front Microbiol. 2022 Aug 30;13:955249. doi: 10.3389/fmicb.2022.955249 (PMC9468665; doi:10.3389/fmicb.2022.955249)
Supplement: Supplementary file 1 [file Data_Sheet_1.PDF]

Supplementary material for

Bromate reduction by *Shewanella oneidensis* MR-1 is mediated by dimethylsulfoxide reductase

Yicheng Wang[1†], Jiale Fan[1†], Yonglin Shen[1], Fan Ye[1], Zhiying Feng[1], Qianning Yang[1], Dan Wang[1], Xunchao Cai[1,2], and Yanping Mao[1*]

[1]College of Chemistry and Environmental Engineering, Shenzhen University, Shenzhen, Guangdong, P.R. China

[2]Department of Gastroenterology and Hepatology, Shenzhen University General Hospital, Shenzhen, Guangdong, P.R. China

*Corresponding author: Yanping Mao; College of Chemistry and Environmental Engineering, Shenzhen University, Shenzhen, Guangdong, P. R. China; Tel: +86-755-26558094; E-mail address: maoy@szu.edu.cn.

[†]These authors have contributed equally to this work and share first authorship.

Submitted to Frontiers in Microbiology

The supplemental material contains 3 tables and 4 figures.

TABLE S1 Composition of trace elemental solution.

Chemical	per liter contained (g)
H_3BO_3	0.50
$CaCl_2$	1.15
$CoCl_2 \cdot 6H_2O$	0.50
$CuSO_4 \cdot 5H_2O$	0.50
$FeSO_4 \cdot 7H_2O$	0.80
$MgSO_4$	4.80
$MnCl_2 \cdot 4H_2O$	0.10
$Na_2EDTA \cdot 2H_2O$	6.00
$Na_2MoO_4 \cdot 2H_2O$	0.40
Na_2SeO_3	0.10
$NiSO_4 \cdot 6H_2O$	0.50
$ZnSO_4 \cdot 7H_2O$	0.20

22 **TABLE S2** Primers used in this study.

Primer	Sequence (5'-3')
HG-VE-1	TGTTCTTTACGATGCCATTGGGAT
HG-VE-2	CGATTACTTCGCCAACTATTGCGA
HG-CH-1	ACGGAAGATCACTTCGCAGAATAAA
HG-CH-2	TTAAGCGTGCATAATAAGCCCTACA
pTac-V1	GAGCTCCATGAATTCTCTCCTGTGT
pTac-V2	AACGCCGTAGCGCCGATGGTAGT
pTac-C1	CACCTCGCTAACGGATTCACC
pTac-C2	CCAATACGCAAACCGCCTCT
Δ*napA*	
Na-UP-1	ATGGCATCGTAAAGAACATGACCATTTCTGCGTCAATGCG
Na-UP-2	TTGCATCCAACCACTTGATATTATCTGACTCGGCGG
Na-DN-1	TATCAAGTGGTTGGATGCAACCGATCCACTGTCAA
Na-DN-2	TAGTTGGCGAAGTAATCGCCAGAGCGGTCGGTAAGACTT

napA-LF	CACTGCCGGCCGTGGTTCTATTA
napA-SR	TTGACAGTGGATCGGTTGCATCCAA
napA-SF	AGGCCATTCTCGATAATGTTGAAG
napA-LR	ATAAGTGTCATGCTCTGCGGATT
Δ*fccA*	
Fc-UP-1	ATGGCATCGTAAAGAACACAACTCAGGCTTGTAGTTGGCG
Fc-UP-2	AGATAGCGTTTACTTCTGGAGCGGCATAGGCTGTGC
Fc-DN-1	TCCAGAAGTAAACGCTATCTCTGATATCGTCACCTA
Fc-DN-2	TAGTTGGCGAAGTAATCGAGCACCTTAATGGTTGCTACTC
fccA-LF	TGCGATGCACTATCGAATAATGCCT
fccA-SR	AGGTGACGATATCAGAGATAGCGTT
fccA-SF	CACAGCCTATGCCGCTCCAGAAGTA
fccA-LR	TCGAGCGCTTCTACCGCTGGATTA
Δ*sye4*	
Sy-UP-1	ATGGCATCGTAAAGAACATTGTTGGCTAATTGTTGCAATA
Sy-UP-2	ATAAGCCTTATAAGGTAATGGTGTCATTAAGCTTGT

Sy-DN-1	CATTACCTTATAAGGCTTATATCGTAACGCTGATAA
Sy-DN-2	TAGTTGGCGAAGTAATCGAATCAATGCATTATCTGTTGTT
sye4-LF	GAAGGTGAATGCAAGATATTGAAC
sye4-LR	GACGCTAATCAATACTCACGATAA
sye4-SF	GCCGGAGATTCTACCTAATATTG
sye4-SR	TCAGCGTTACGATATAAGCCTTA
Δ*cymA*	
Cy-UP-1	ATGGCATCGTAAAGAACAAACTTGAGCTCGTAATCTGCT
Cy-UP-2	CAGCGTTAAACCTTATCCACGCCAGTTCATTACTCTATCT
Cy-DN-1	AGAGTAATGAACTGGCGTGGATAAGGTTTAACGCTGCAAT
Cy-DN-2	TAGTTGGCGAAGTAATCGCTAAGCCATATACCGCTGAAGT
cymA-LF	CATTGTGCTAATGATGAATGATGC
cymA-SF	GCATAGCTCTCACTATCGTCGATT
cymA-SR	TATTGCAGCGTTAAACCTTATCC
cymA-LR	ACCAAGGTGTTGGCCATTAATAAC
Δ*gspD*	

Gs-UP-1	ATGGCATCGTAAAGAACATCGATCTCGATGGATTACAGCA
Gs-UP-2	TCCACTCATCTCCAGCAATCAGTTTGCGTCGAATCC
Gs-DN-1	GATTGCTGGAGATGAGTGGAACCAGTCTGAGTACTT
Gs-DN-2	TAGTTGGCGAAGTAATCGATATCGAGGCGCGCCATAACCT
gspD-LF	GAAGCTCGTACCTACCTCATCTTCT
gspD-SR	AGTACTCAGACTGGTTCCACTCATC
gspD-SF	GATTCGACGCAAACTGATTGCTGGA
gspD-LR	CGTGGCTCGATGGCATGGTCGATAC
Δ*mtrB*	
MB-UP-1	ATGGCATCGTAAAGAACAAAGGTGTCCACGGTGCGATTGA
MB-UP-2	GGCTGTTACTTGTGTTGGCTAATAACGCTAGAGTGA
MB-DN-1	AGCCAACACAAGTAACAGCCATGACTACACCGCACA
MB-DN-2	TAGTTGGCGAAGTAATCGTGCTCACGACTGACATTTAGCC
mtrB-LF	AAGGCGCCGATTCTTGCTTGATGTG
mtrB-SR	TGTGCGGTGTAGTCATGGCTGTTAC
mtrB-SF	TCACTCTAGCGTTATTAGCCAACAC

mtrB-LR	TCCATGGCATATTGGCCGAGTACTA
Δ*mtrC*	
MC-UP-1	ATGGCATCGTAAAGAACAACCGATTGTGCAACATGTCATA
MC-UP-2	CAACTGTTGGCATTGTGACGGCACTTGCTGCGAGCA
MC-DN-1	CGTCACAATGCCAACAGTTGCAGATCACACTAAAGT
MC-DN-2	TAGTTGGCGAAGTAATCGACAGGTATCATTCACGCTAGGC
mtrC-LF	CAGGCGTACAATCTGGCACTGTACT
mtrC-SR	ATTGCCATTGTGATGGTAAGTGCCG
mtrC-SF	GCTCGCAGCAAGTGCCGTCACAAT
mtrC-LR	CAAGTGACACAATTCTCAGTGACGG
Δ*dmsA*	
Dm-UP-1	ATGGCATCGTAAAGAACATTATCTCGCAGACCGTGAACGT
Dm-UP-2	TGTGTTGTGGATTCACATCACTAGAACCTGAGTTAC
Dm-DN-1	TGATGTGAATCCACAACACACCAATCGTGTGCAGAT
Dm-DN-2	TAGTTGGCGAAGTAATCGCACTACCACCAACTGGTTGACC
dmsA-LF	CAGCGGCTCTGTCTACAACGCTGT

dmsA-SR	TCTGCACACGATTGGTGTGTTGTGG
dmsA-SF	CCTGCTGCCTTGATTGATAAGAGGT
dmsA-LR	GCCACTGGCTTGCTAACTCATCTCG
C-Δ*cymA*	
cymA-CF	AGAGAATTCATGGAGCTCGTAATGAACTGGCGTGCACTAT
cymA-CR	CATCGGCGCTACGGCGTTTGCAGCGTTAAACCTTATCCTT
C-Δ*dmsA*	
dmsA-CF	AGAGAATTCATGGAGCTCAGACAATGGAACGCAGAAGTT
dmsA-CR	CATCGGCGCTACGGCGTTCTTATGCCTTGACGATCTGCAC

23

24

TABLE S3 Reference genome information for 57 *Shewanella* species downloaded from the NCBI database.

Organism Name	Assembly Name	Assembly Accession	Assembly Level
Shewanella aestuarii	ASM1464721v1	GCF_014647215.1	Contig
Shewanella algae	ASM973065v1	GCF_009730655.1	Complete Genome
Shewanella algicola	ASM1464803v1	GCF_014648035.1	Contig
Shewanella algidipiscicola	ERP106791_PRJEB24924	GCF_900380485.1	Contig
Shewanella amazonensis	ASM1524v1	GCF_000015245.1	Complete Genome
Shewanella atlantica	ASM396626v1	GCF_003966265.1	Scaffold
Shewanella baltica	ASM17887v2	GCF_000178875.2	Complete Genome
Shewanella benthica	ASM1506987v1	GCF_015069875.1	Scaffold
Shewanella bicestrii	ASM221687v1	GCF_002216875.1	Complete Genome
Shewanella canadensis	ASM396622v1	GCF_003966225.1	Contig
Shewanella carassii	ASM1967070v1	GCF_019670705.1	Complete Genome
Shewanella chilikensis	ASM283694v1	GCF_002836945.1	Scaffold
Shewanella colwelliana	ASM173552v1	GCF_001735525.1	Scaffold
Shewanella corallii	ASM335308v1	GCF_003353085.1	Contig
Shewanella decolorationis	ASM435430v1	GCF_004354305.1	Chromosome
Shewanella denitrificans	ASM1376v1	GCF_000013765.1	Complete Genome
Shewanella dokdonensis	ASM1839433v1	GCF_018394335.1	Complete Genome
Shewanella donghaensis	ASM756750v1	GCF_007567505.1	Complete Genome

Shewanella fidelis	ASM51860v1	GCF_000518605.1	Scaffold
Shewanella fodinae	ASM434240v1	GCF_004342405.1	Scaffold
Shewanella frigidimarina	ASM1470v1	GCF_000014705.1	Complete Genome
Shewanella gelidii	ASM1464785v1	GCF_014647855.1	Scaffold
Shewanella glacialipiscicola	ASM1965523v1	GCF_019655235.1	Contig
Shewanella hafniensis	ASM1965533v1	GCF_019655335.1	Contig
Shewanella halifaxensis	ASM1918v1	GCF_000019185.1	Complete Genome
Shewanella hanedai	ASM719764v1	GCF_007197645.1	Contig
Shewanella indica	ASM283697v1	GCF_002836975.1	Scaffold
Shewanella insulae	ASM983165v1	GCF_009831655.1	Contig
Shewanella intestini	ASM1822146v1	GCF_018221465.1	Scaffold
Shewanella inventionis	ASM1464185v1	GCF_014641855.1	Scaffold
Shewanella japonica	ASM207579v1	GCF_002075795.1	Complete Genome
Shewanella khirikhana	ASM395774v1	GCF_003957745.1	Complete Genome
Shewanella litoralis	ASM1464833v1	GCF_014648335.1	Scaffold
Shewanella litorisediminis	ASM1683445v1	GCF_016834455.1	Complete Genome
Shewanella livingstonensis	ASM385539v1	GCF_003855395.1	Complete Genome
Shewanella loihica	ASM1606v1	GCF_000016065.1	Complete Genome
Shewanella mangrovi	ASM75379v1	GCF_000753795.1	Contig
Shewanella marina	ASM61497v1	GCF_000614975.1	Contig

Shewanella marisflavi	ASM221558v1	GCF_002215585.1	Complete Genome
Shewanella maritima	ASM429534v1	GCF_004295345.1	Complete Genome
Shewanella morhuae	IMG-taxon 2681812898 annotated assembly	GCF_900156405.1	Scaffold
***Shewanella oneidensis* MR-1**	**ASM14616v2**	**GCF_000146165.2**	**Complete Genome**
Shewanella pealeana	ASM1828v1	GCF_000018285.1	Complete Genome
Shewanella piezotolerans	ASM1488v1	GCF_000014885.1	Complete Genome
Shewanella polaris	ASM638555v1	GCF_006385555.1	Complete Genome
Shewanella psychrophila	ASM200530v1	GCF_002005305.1	Complete Genome
Shewanella putrefaciens	ASM1640632v1	GCF_016406325.1	Complete Genome
Shewanella sairae	ASM1965501v1	GCF_019655015.1	Contig
Shewanella saliphila	ASM1464829v1	GCF_014648295.1	Scaffold
Shewanella schlegeliana	ASM1676563v1	GCF_016765635.1	Scaffold
Shewanella sediminis	ASM1802v1	GCF_000018025.1	Complete Genome
Shewanella ulleungensis	ASM1464831v1	GCF_014648315.1	Contig
Shewanella vesiculosa	ASM379788v1	GCF_003797885.1	Scaffold
Shewanella violacea	ASM9132v1	GCF_000091325.1	Complete Genome
Shewanella waksmanii	ASM51880v1	GCF_000518805.1	Scaffold
Shewanella woodyi	ASM1952v1	GCF_000019525.1	Complete Genome
Shewanella xiamenensis	ASM313054v1	GCF_003130545.1	Contig

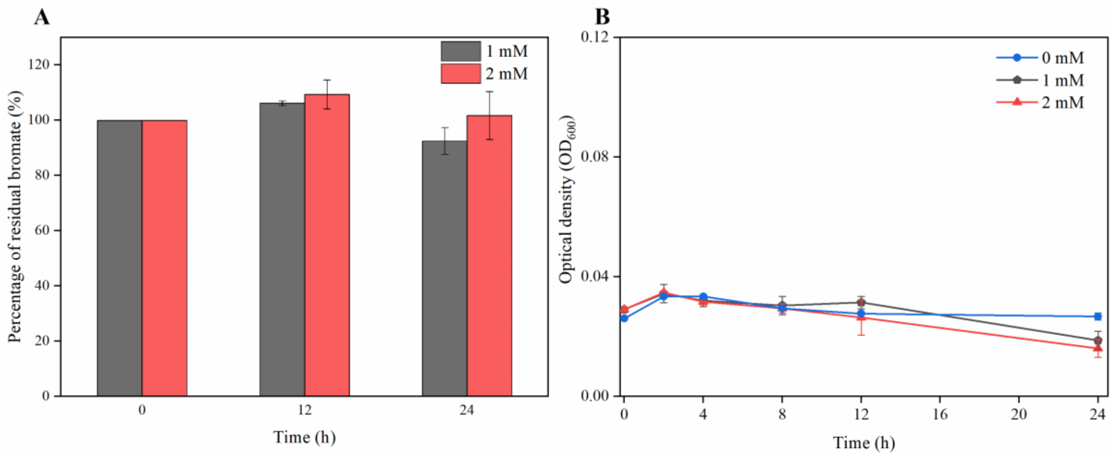

FIGURE S1 Anaerobic bromate reduction by *S. oneidensis* MR-1. (A) The strain was cultured with the dosage of bromate at 1 mM and 2 mM, respectively. (B) Biomass changes of the strain during anaerobic reduction of bromate at 0 mM, 1 mM, and 2 mM. Error bars represent standard deviations of triplicate samples.

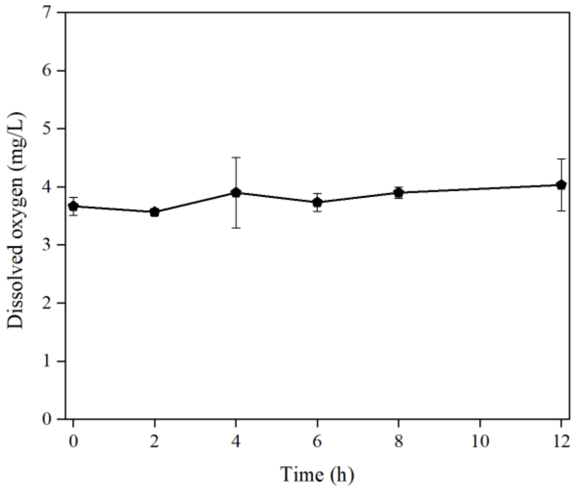

FIGURE S2 The dissolved oxygen (DO) concentration in cultures. Error bars represent standard deviations of triplicate samples.

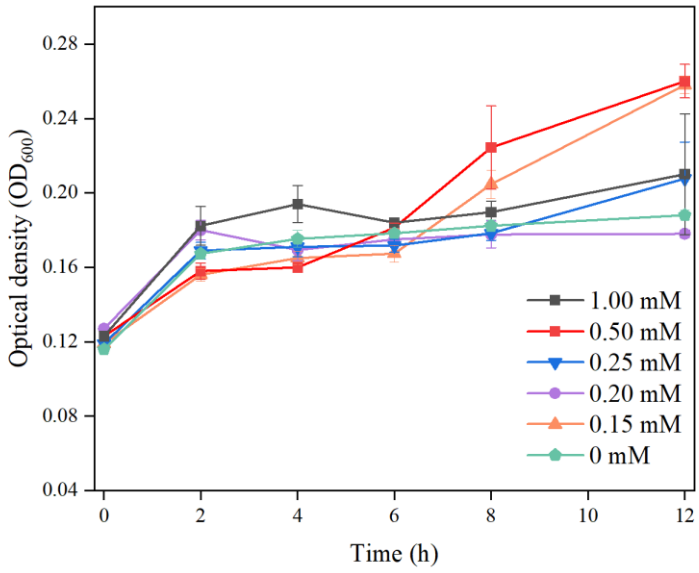

FIGURE S3 Biomass changes of *S. oneidensis* MR-1 during microaerobic bromate reduction. The strain was cultured with the dosage of bromate at 0 mM, 0.15 mM, 0.20 mM, 0.25 mM, 0.50 mM, and 1.00 mM respectively. Error bars represent standard deviations of triplicate samples.

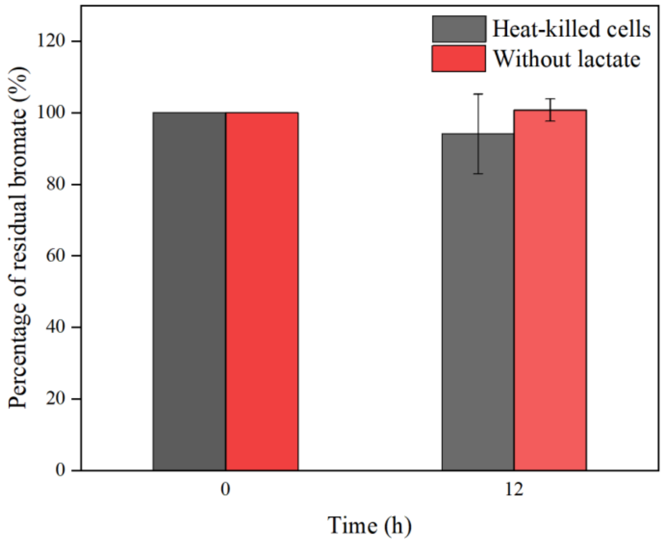

FIGURE S4 Microaerobic bromate reduction using heat-killed cells or without lactate. The initial dosage of bromate was 0.25 mM. Error bars represent standard deviations of triplicate samples.
